# Fractionated testosterone gel replacement therapy in clinical practice: A real-world exploratory clinical experience

**DOI:** 10.1007/s12020-026-04659-8

**Published:** 2026-05-23

**Authors:** Virginia Maltese, Andrea Delbarba, Elisa Gatta, Simone Vetrugno, Andrea Sansone, Carlo Cappelli

**Affiliations:** 1 Department of Clinical and Experimental Sciences, Endocrine and Metabolic Unit, University of Brescia, Azienda Socio-Sanitaria Territoriale (ASST) Spedali Civili di Brescia, Brescia, Italy; 2 Department of Internal Medicine and Therapeutics, University of Pavia, Pavia, Italy; 3 Chair of Endocrinology and Sexual Medicine (ENDOSEX), Department of Systems Medicine, University of Rome Tor Vergata, Rome, Italy

**Keywords:** testosterone replacement therapy, male hypogonadism, transdermal testosterone gel, dose fractionation

## Abstract

**Background:**

Testosterone replacement therapy (TRT) with transdermal gel is widely used in men with hypogonadism. Guidelines recommend once-daily application with monitoring of serum testosterone at peak levels. However, real-world clinical experience suggests that peak–trough fluctuations in some patients, potentially affecting hormonal stability and treatment tolerability. The impact of dose fractionation remains unclear.

**Objectives:**

To compare hormonal profiles and biochemical safety parameters between once-daily and twice-daily (fractionated) testosterone gel administration in hypogonadal men receivingh the same total daily dose.

**Methods:**

We retrospectively identified hypogonadal men treated with 2% testosterone gel who transitioned from once-daily to twice-daily dosing, maintaining the same total daily dose. Eligible patients had serum total testosterone (TT) measurements available at both peak (3 h post-application) and nadir (pre-dose). Hormonal and laboratory parameters under the two regimens were compared.

**Results:**

Twelve patients met inclusion criteria. Fractionated dosing was associated with lower peak TT levels [9.0 (5.3–13.0) vs. 5.7 (3.6–7.1) ng/mL, paired difference − 3.6 ng/mL (95% CI: −4.9 to − 1.8)] and higher nadir TT levels [1.4 (1.1–1.8) vs. 3.2 (2.6–5.4) ng/mL, paired difference 2.6 ng/mL (95% CI: 1.2 to 5.1)], resulting in a reduced difference between peak-to-pre-dose difference within the observed sampling window. The proportion of patients achieving target TT levels at peak increased (33% vs. 83%). Calculated free testosterone showed a similar pattern. No clinically relevant differences were observed in luteinizing hormone, prostate-specific antigen, haematocrit, or haemoglobin.

**Conclusion:**

In this real-world cohort, fractionated testosterone gel administration was associated with a higher proportion of patients achieving biochemical targets and with a reduced peak-to-pre-dose difference within the observed sampling window, without short-term biochemical safety signals. These findings should be considered hypothesis-generating.

## Introduction

Male hypogonadism is a clinical syndrome characterized by the inability of the testes to produce physiological concentrations of testosterone and/or a normal sperm count [1, 2]. The diagnosis is based on the finding of low morning serum total testosterone (TT) levels in association with typical symptoms and signs of androgen deficiency [1–5], which include, but are not limited to, erectile dysfunction, decreased libido, loss of morning erections, and fatigue.

The prevalence of hypogonadism increases with age [6, 7], as aging is associated with a gradual decline in testosterone concentrations. Among men aged 40 to 79 years, the prevalence of symptomatic hypogonadism ranges from 2.1% to 5.7%, with approximately 11.7–12.3 new cases per 1,000 individuals per year [8].

Testosterone replacement therapy (TRT) is the standard of care for most patients, aiming to restore physiological serum testosterone concentrations and thereby improve hypogonadal symptoms and quality of life [1, 9]. TRT is considered both safe [4, 10, 11] and effective [9] when appropriately monitored. Several formulations are available—including injectable preparations, transdermal gels, patches, subcutaneous pellets, and oral tablets—and the choice depends on pharmacokinetic profile, cost, comorbidities, and, most importantly, patient preference and adherence [2].

Among these, transdermal testosterone gel is the most widely prescribed formulation [4, 12]. After topical application, serum testosterone typically peaks within 2–4 h, followed by a gradual decline over 4–6 h, with a low risk of supraphysiological levels [13].

The harmonized reference interval for TT in healthy, non-obese men aged 19–39 years is 10.5–29.5 nmol/L (3.03–8.52 ng/mL) when defined by the 5th and 95th percentiles, or 9.2–31.8 nmol/L (2.64–9.16 ng/mL) when defined by the 2.5th and 97.5th percentiles [14]. Several guidelines have proposed different thresholds for the biochemical diagnosis of hypogonadism [1–4]: the Italian Society of Andrology and Sexual Medicine, in its clinical practice guidelines [2], suggests using a threshold of 12 nmol/L, based on meta-analysis studies [15, 16] which showed no efficacy for TRT above these values. However, a universal agreement on the suggested threshold for diagnosis has not been reached. Likewise, target TT levels during TRT are not yet universally standardized. However, most guidelines recommend maintaining serum TT within the mid-normal range of healthy young men [1–5].

In our clinical practice, we consider a TT threshold of 3.5 ng/mL as the lower acceptable limit during TRT. To assess treatment adequacy, guidelines recommend measuring TT levels 2–4 h [3] (or 2–8 h [1]) after gel application, corresponding to the expected pharmacokinetic peak.

Transdermal gel therapy is generally well tolerated. The most common adverse effects are local skin reactions—burning, tingling, dryness, erythema, or itching—which usually decrease or resolve with continued use [17, 18]. However, many patients fail to maintain stable concentrations throughout the day following application of TRT gel. This is unsurprising, since pharmacokinetic data from clinical trials rarely apply to the real-world setting, and as such several factors can affect not only the absorption rate through the skin, but also individual metabolism of the drug itself. In a recent study, in order to better evaluate overall exposure to TRT, the authors proposed measuring TT 23 h post-application, just before the next dose, in patients who achieved adequate TT levels 2 h after application [18]. This approach was also employed in a large multicentre American randomized controlled trial (TRT gel vs. placebo) involving 5,264 hypogonadal men, which found that assessing TT 24 h post-application was clinically useful for treatment adjustment [10]. We frequently apply this method in our clinical practice, particularly for older men or patients with demanding work schedules.

During routine clinical practice, we observed a patient who had independently modified his prescribed once-daily testosterone gel regimen by splitting the dose into two applications (morning and evening) to alleviate local skin reactions. Following this observation, we identified 11 additional patients who had adopted the same twice-daily administration schedule while maintaining an identical total daily dose. The primary aim of this study was to compare serum TT levels between patients treated with the standard once-daily regimen and those using a twice-daily split-dose schedule. Given the real-world and self-initiated nature of the dosing modification, the present study should be considered exploratory and hypothesis-generating.

## Materials and methods

### Patients

Patients were recruited by searching the database of hypogonadal subjects on TRT treatment followed up at the Andrology outpatient service of the Endocrinology Unit, ASST Spedali Civili of Brescia. The search criteria were as follows: (1) treatment with 2% testosterone gel (Tostrex^®^, Advanz Pharma Italia S.r.l., Italy) administered at a daily dose of 5 actuations (50 mg/day); (2) spontaneous transition from once-daily to twice-daily application (morning and evening) while maintaining the same total daily dose; (3) availability of serum TT measurements at both peak and nadir before and after regimen modification.

All patients had been receiving stable once-daily testosterone gel therapy for at least 6 months before switching regimen, ensuring steady-state conditions. Laboratory evaluations under the fractionated regimen were performed after a minimum of 6 months of stable twice-daily administration. This approach ensured that all measurements reflected steady-state conditions under both dosing regimens. Formal adherence measures were not available due to the retrospective design; however, adherence and treatment continuity were routinely assessed during outpatient visits through structured patient questioning, in accordance with good clinical practice.

Peak measurements were obtained 3 h after the morning application, and nadir samples immediately before the subsequent dose, according to standardized clinical practice instructions.

The study was approved by the Local Ethics Committee (N. 6182).

### Laboratory assessments

Laboratory tests were performed in all patients according to standard clinical practice. All blood samples were collected in the morning after an overnight fast. Blood samples were collected to evaluate total testosterone (TT: normal range: 2.49–8.36 ng/mL; limit of quantitation 0.12 ng/mL), luteinizing hormone (LH; normal range: 1.7–8.6 IU/L; limit of quantitation 1 mIU/mL), haematocrit (HCT; normal range: 42–52%), haemoglobin (Hb; normal range for male patients: 14–18 g/dL), and prostate-specific antigen (PSA; normal range: <1.4 mcg/L) were performed. Circulating free testosterone (cFT) was calculated based on total testosterone, sex hormone-binding globulin (SHBG; normal range: 18.8–54.1 nmol/L; limit of detection 0.35 nmol/L), and albumin (normal range: 3.1–5.2 g/dL) levels according to the Vermeulen formula [19, 20]. All measurements were performed after at least 6 months of stable treatment under each dosing regimen. Serum TT levels were collected at both peak (3 h after gel application) and nadir (immediately before the subsequent application). Patients were classified as achieving therapeutic targets when peak TT values fell within the 3.5–9 ng/mL range [4]. This interval was used as a pragmatic classification tool to distinguish under-, within-, and over-treatment categories. In particular, the upper threshold (9 ng/mL) was not intended as a therapeutic target but rather to identify values approaching the upper limit of the physiological reference range, thus indicating clearly supraphysiological exposure at peak.

### Statistical analysis

Data are presented as median (interquartile range) for continuous variables and as categorical variables when appropriate. Normality was assessed using the Shapiro–Wilk test. Continuous variables were compared using the Mann–Whitney U test for independent samples and the Wilcoxon signed-rank test for paired samples, as appropriate. Categorical variables were compared using the chi-square test or Fisher’s exact test, as applicable. To assess agreement between paired categorical evaluations across more than two outcome levels (testosterone target: under-treated, within target, and over-treated), Bowker’s test of symmetry was applied. Paired differences for the main hormone outcomes were further summarized using the Hodges–Lehmann estimator with corresponding 95% confidence intervals derived from the Wilcoxon signed-rank test. Given the small sample size, p-values were interpreted cautiously and considered exploratory.

All statistical analyses were performed using SPSS version 20.0 (SPSS Inc., Evanston, IL, USA) and RStudio version 4.2.2.

## Results

Among 262 hypogonadal patients on TRT, 12 (4.6%) subjects fulfilled the inclusion criteria and were recruited in the study. Overall, 9 (75%) patients were affected by primary hypogonadism, including 5 (42%) subjects with history of bilateral orchiectomy, 2 (17%) with non-specific testicular damage, 1 (8%) with Klinefelter syndrome, and one (8%) with cryptorchidism. Among 3 (25%) patients with hypogonadotropic hypogonadism, one (8%) had a pituitary adenoma whereas 2 (17%) had no identifiable organic cause on pituitary imaging. The clinical and demographic data are reported in Table 1.

**Table 1:** Baseline clinical characteristics of the study population

Age (years)	46 (42–51)
Weight (Kg)	86 (75–92)
Height (cm)	178 (172–190)
BMI (Kg/m^2^)	25.9 (24.4–28.2)
Years of hypogonadism	4 (2–16)

BMI: body mass index. Data are expressed as median (IQR)

**Fig. 1:**
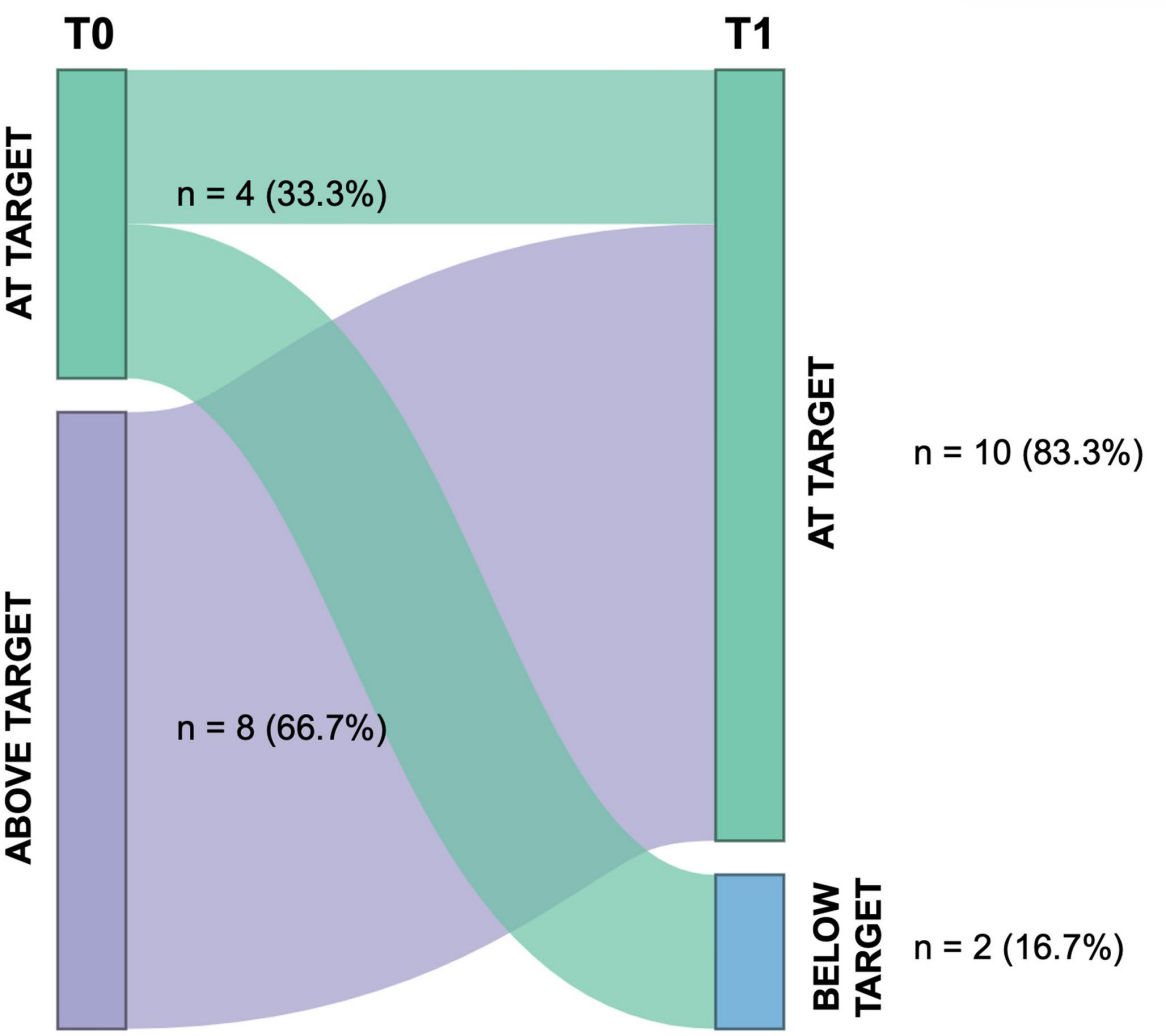
Sankey diagram illustrating transitions between testosterone target categories from once-daily (T0) to twice-daily (T1) administration, measured at peak

During treatment with once-daily testosterone gel, 33% of patients (*n* = 4) achieved therapeutic target of TT levels, whereas 67% showed values approaching or exceeding the upper limit of the reference range at peak, consistent with potential pharmacological overdosing (*n* = 8). After splitting, 83% achieved target (*n* = 10), whereas 17% were in pharmacological underdosing (*n* = 2) at peak (*p* = 0.019) (Fig. 1).

In depth, at peak, TT levels decreased after dose splitting [9.0 (5.3–13.0) vs. 5.7 (3.6–7.1) ng/mL], with a Hodges–Lehmann paired difference of − 3.6 ng/mL (95% CI: −4.9 to − 1.8). At nadir, TT levels increased [1.4 (1.1–1.8) vs. 3.2 (2.6–5.4) ng/mL], with a paired difference of 2.6 ng/mL (95% CI: 1.2 to 5.1). Similar directional changes were observed for cFT. At peak, cFT levels tended to decrease [93.6 (87.9–97.2) vs. 78.7 (59.1–92.8) ng/L], with a paired difference of -16.7 (95% CI: -37.9 to -1.2). At nadir, cFT levels increased [27.9 (21.1–34.5) vs. 88.3 (76.4–158.3) ng/L], with a paired difference of 87.6 ng/L (95% CI: 40.7 to 159.8). All other biochemical parameters remained unchanged before and after the modification of the dosing regimen (Table 2), including serum LH [2.6 (0.4–12.2) vs. 1.2 (0–2.9) IU/L, *p* = 0.374], PSA [0.9 (0.6–1.1) vs. 0.8 (0.5–1.0) mcg/L, *p* = 0.120] and HCT [47.0 (44.0–48.0) vs. 47.8 (46.0–48.1) %, *p* = 0.753].

**Table 2:** Hormonal and laboratory parameters during once-daily and twice-daily testosterone replacement therapy

	TRT One-daily dose	TRT Twice-daily dose	HL paired difference (95% CI)	*p*
TT (ng/mL) at peak	9.0 (5.3–13.0)	5.7 (3.6–7.1)	-3.6 (95% CI: -4.9 to -1.8)	0.002
cFT (ng/L) at peak	93.6 (87.9–97.2)	78.7 (59.1–92.8)	-16.7 (95% CI: -37.9 to -1.2)	0.048
TT (ng/mL) at nadir	1.4 (1.1–1.8)	3.2 (2.6–5.4)	2.6 (95% CI: 1.2 to 5.1)	0.005
cFT (ng/L) at nadir	27.9 (21.1–34.5)	88.3 (76.4–158.3)	87.6 (95% CI: 40.7 to 159.8)	0.018
LH (IU/L)	2.6 (0.4–12.2)	1.2 (0–2.9)	-	0.374
PSA (mcg/l)	0.9 (0.6–1.1)	0.8 (0.5–1.0)	-	0.120
HCT (%)	47.0 (44.0–48.0)	47.8 (46.0–48.1)	-	0.753
Hb (g/dl)	16 (15.8–16.7)	16.2 (15.8–16.4)	-	0.720

TRT: testosterone replacement therapy; HL: Hodges–Lehmann; CI: confidence interval; TT: total testosterone; cFT: calculated free testosterone; LH: luteinizing hormone; PSA: prostate-specific antigen; HCT: haematocrit; Hb: haemoglobin. Data are expressed as median (IQR)

Hodges–Lehmann paired differences with 95% confidence intervals are reported for the main hormone outcomes; p-values are exploratory due to small sample size

**Fig. 2:**
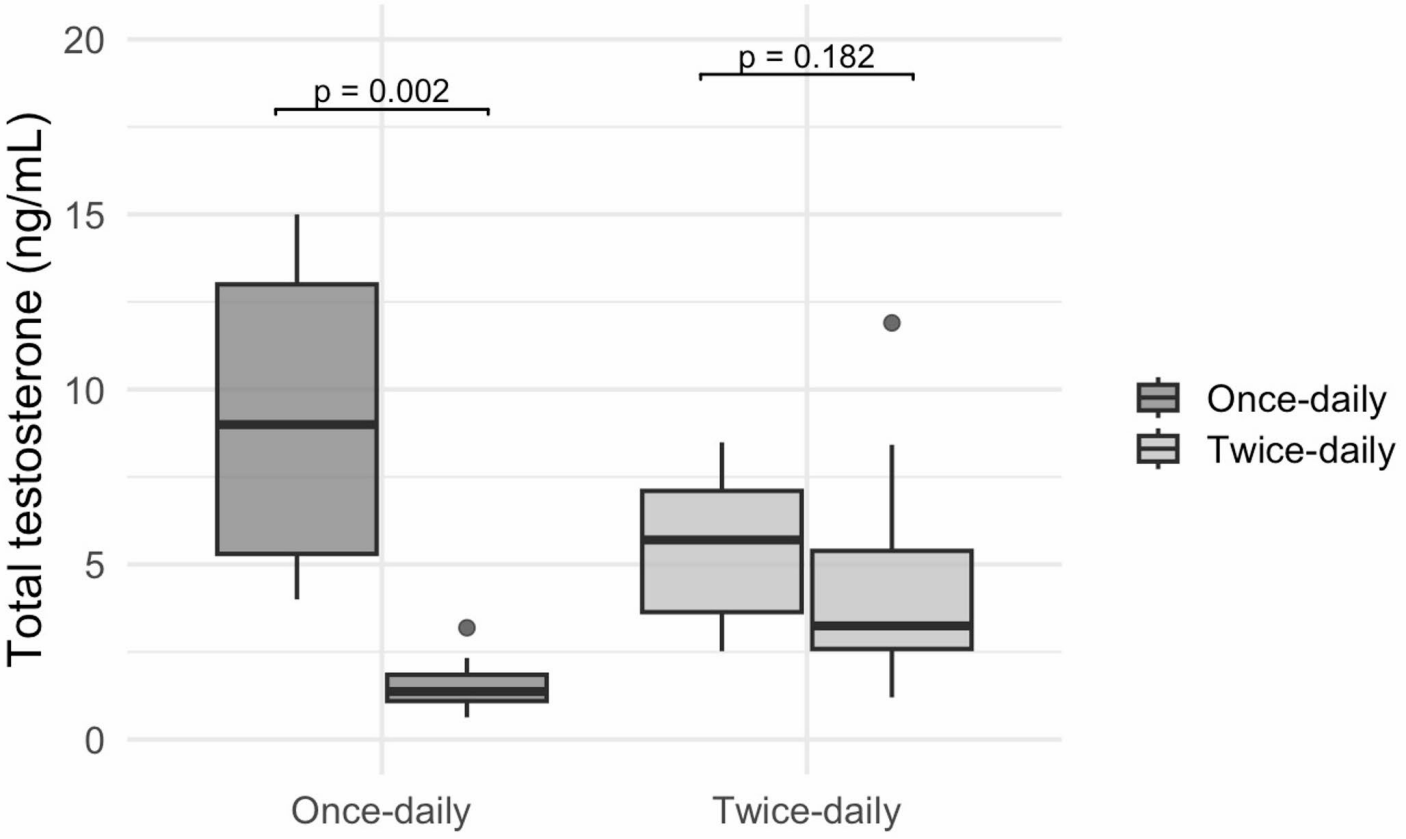
Box-plot illustrating total testosterone target serum level at peak and nadir, on once-daily and twice-daily administration

Under once-daily TRT, a marked difference between peak and nadir TT levels was observed [9.0 (5.3–13.0) vs. 1.4 (1.1–1.8) ng/mL], with a paired difference of − 7.5 ng/mL (95% CI: −10.4 to − 4.8). In contrast, this difference was attenuated within the observed sampling window after dose splitting [5.7 (3.6–7.1) vs. 3.2 (2.6–5.4) ng/mL; paired difference − 1.3 ng/mL (95% CI: −2.9 to 1.0)] (Fig. 2).

**Fig. 3:**
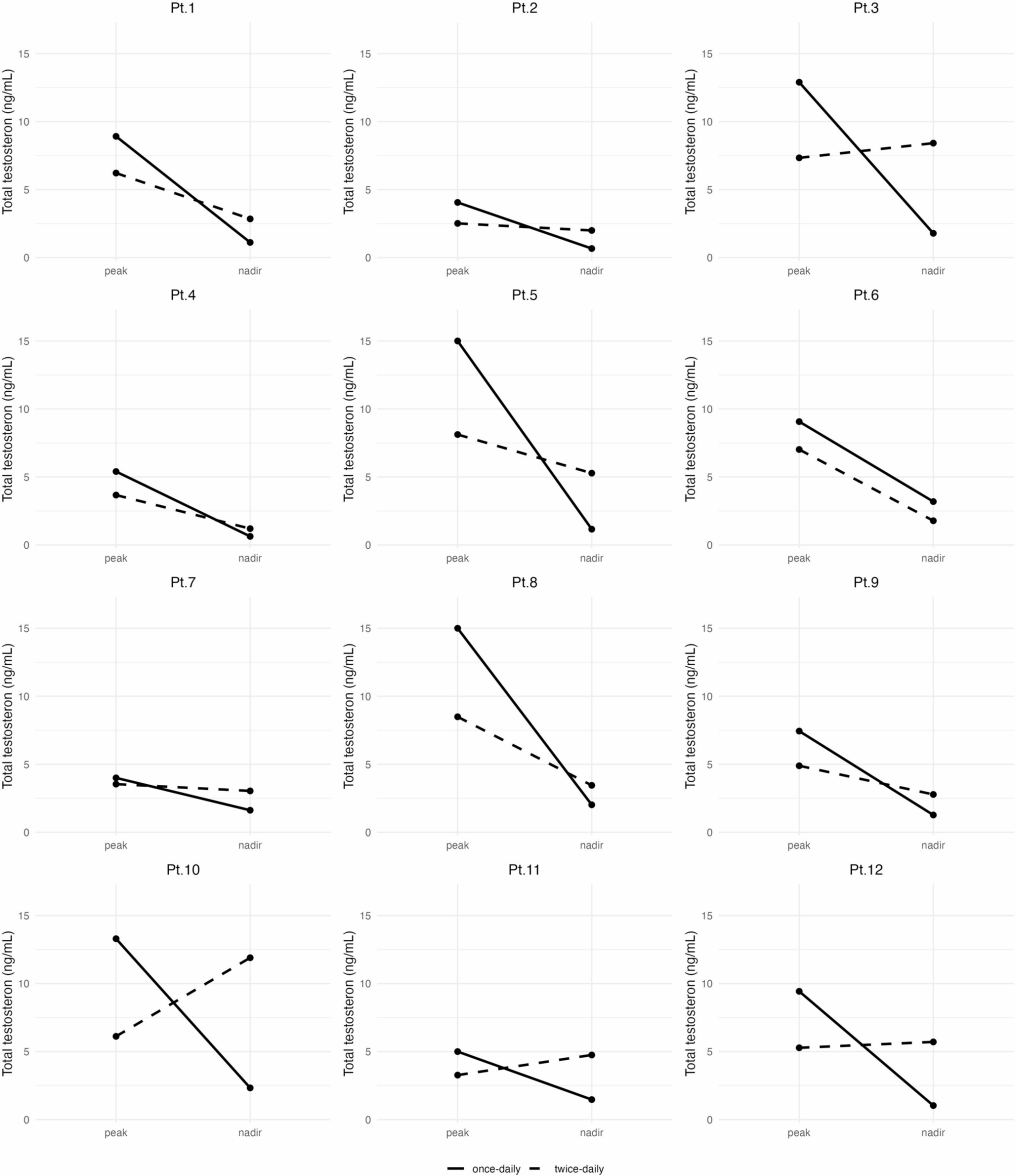
Individual trajectories of total testosterone (TT) at peak and nadir under once-daily vs. twice-daily testosterone gel regimens. Solid lines depict TT values obtained during once-daily administration, while dashed lines represent TT values during twice-daily fractionated dosing

The TT levels variations in each patient are reported in Fig. 3.

## Discussion

In this real-world, non-randomized cohort, fractionated testosterone gel administration was associated with improved biochemical target attainment in this selected cohort and with a reduced difference between peak and pre-dose testosterone concentrations within the observed sampling window, while maintaining total daily dosage unchanged.

It should be emphasized that the upper threshold used in our classification (9 ng/mL) does not reflect an intended therapeutic target. In line with current guidelines, optimal testosterone replacement therapy aims to maintain serum testosterone within the mid-normal range. In our study, this upper limit was used solely to identify values approaching the upper boundary of the physiological reference range, thus indicating clearly supraphysiological exposure at peak. Notably, fractionated dosing shifted testosterone concentrations toward the mid-normal range, thereby improving alignment with guideline-based targets. It is important to emphasize that this therapeutic approach has not previously been formally described, and that the most authoritative guidelines continue to recommend a once-daily testosterone gel application [1–5]. Current recommendations also focus exclusively on peak assessment, advising that TT levels be measured 2–8 h after gel application [1, 3], while offering no indications regarding nadir measurements. As a result, the extent to which daily fluctuations in testosterone might influence clinical outcomes remains largely unexplored.

However, emerging evidence has begun to highlight the potential relevance of nadir concentrations. Sansone et al. recently demonstrated that although approximately 70% of patients achieved biochemical normalization at peak, only 37% maintained adequate TT levels at nadir, suggesting that assessment at both time points may provide a more accurate representation of overall hormonal exposure [21]. Moreover, a recent review on the chronobiology of hormone replacement proposed evaluating TT both before and after drug administration to better characterize absorption kinetics and trough values [22]. Such an approach may help clinicians deliver a more individualized and physiologically tailored treatment, possibly minimizing periods of relative androgen deficiency and reducing the likelihood of supraphysiological excursions.

The potential clinical relevance of nadir measurements is further reinforced by the large, multicentre, randomized, double-blind TRAVERSE trial, in which total testosterone was assessed only at approximately 24 h after administration. Notably, despite enrolling men at elevated cardiovascular risk, the study reported no excess in major adverse cardiovascular events among testosterone-treated participants [10]. Although not designed to explore pharmacokinetics, the choice of a 24-hour sampling point implicitly supports the hypothesis that trough levels may meaningfully reflect cumulative exposure.

Within this conceptual framework, our findings suggest that a twice-daily regimen attenuates the magnitude of the peak-to-pre-dose variation observed under once-daily administration. This observation should not be interpreted as evidence of improved pharmacokinetic stability, given the non-comparability of nadir sampling between regimens. However, given the different timing of pre-dose sampling (approximately 24 h after dosing in the once-daily regimen and approximately 12 h after the last application in the twice-daily regimen), the present study cannot determine whether fractionated dosing improves true 24-hour pharmacokinetic stability. Accordingly, the present findings should be interpreted as descriptive of the observed sampling window rather than indicative of full daily pharmacokinetic behaviour.

By dividing the same total testosterone dose into two applications, we observed a pronounced attenuation of peak–trough variability, yielding serum testosterone values that displayed a narrower peak-to-pre-dose range within the observed sampling window (Fig. 2). It is important to acknowledge that the comparison of pre-dose concentrations between regimens reflects measurements obtained at different post-application intervals (approximately 24 h vs. 12 h). Therefore, the observed differences should be interpreted as changes in peak-to-pre-dose variation within the respective dosing schedules, rather than as evidence of improved full 24-hour hormonal stability.

If a fractionated regimen were to be adopted more broadly, circadian physiology should also be considered. Administering a larger proportion of the total dose in the morning may better recapitulate endogenous testosterone rhythms [22], potentially optimizing both biochemical control and symptom relief.

A stable serum TT profile has been associated in previous studies with improved symptom control, lower haematocrit variability, and favourable metabolic and skeletal effects [23–25] . However, the present study was not designed to evaluate clinical outcomes. Therefore, no inference can be made regarding potential symptomatic or long-term safety advantages of dose fractionation. Moreover, twice-daily application may introduce practical challenges, including potential reductions in adherence due to the need for repeated daily administration, as well as a theoretical increase in the window for secondary transference before complete gel absorption. These aspects warrant dedicated prospective investigation.

Importantly, in our series of patients, none of them exposed to the fractionated regimen exhibited supraphysiological testosterone levels when assessed at peak, fully in accordance with current guideline recommendations (Fig. 1). As extensively described, supraphysiological TT levels have been shown to induce endothelial injury through increased generation of reactive oxygen species, ERK1/2 pathway activation, and NLRP3-inflammasome–mediated vascular dysfunction [26–28]. However, the present dataset does not allow any mechanistic inference. Therefore, the potential vascular implications of peak attenuation remain, although very interesting, purely speculative. In fact, given the limited sample size and short duration of observation, no conclusions can be drawn regarding the comparative safety of the two regimens. Rather, our findings indicate the absence of short-term biochemical safety signals within the observed timeframe.

Another potential benefit of divided dosing lies in the reduction of cutaneous adverse effects. In fact, as well evidenced in a very recent review by Chawareb et al. local reactions after gel application have been frequently reported—including burning sensations, tingling, dryness, erythema, rash, or pruritus. The Authors concluded that even if dermatologic side effects of TRT are often considered non-severe, they can significantly impact patient satisfaction, adherence, and quality of life [18].

Although these effects generally diminish with continued therapy [17, 18], administering smaller gel quantities twice daily may help reduce the intensity of such reactions and improve overall tolerability. Prospective studies are needed to clarify this dermatological issue.

Nonetheless, we must emphasize that the interpretation of these case-based data, even if well documented, should be approached with caution.

Further studies—preferably randomized, adequately powered, and incorporating structured timing of TT assessment—are needed to determine the true clinical relevance of twice-daily testosterone gel administration. Future research should evaluate not only biochemical indices but also patient-reported outcomes (i.e. quality of life), metabolic parameters and cardiovascular biomarkers. Identifying predictors of peak–nadir variability—including hepatic metabolic capacity, skin absorption kinetics, and SHBG-related differences—will be essential for recognizing patients who may benefit most from this therapeutic approach.

The present study has several important limitations. First, its retrospective design and the self-initiated dosing modification introduce potential selection and behavioural bias. Patients who independently adopted fractionated dosing may represent a more motivated or treatment-aware subgroup, possibly influencing adherence and monitoring frequency. Importantly, the self-selected adoption of fractionated dosing may identify a subgroup of patients with higher motivation, treatment awareness, or intolerance to standard regimens, thus limiting the generalizability of the findings. Additionally, adherence was not formally assessed, which may represent a further source of variability in real-world treatment exposure. Second, the timing of laboratory assessments, although performed according to routine clinical practice, was not prospectively standardized in relation to exact application timing under controlled conditions. This limitation should be considered central when interpreting the pharmacokinetic implications of our findings. Third, the small sample size limits statistical power and precludes adjustment for potential confounders. The cohort was heterogeneous in terms of underlying aetiology of hypogonadism, including both primary and secondary forms. This heterogeneity further limits the generalizability of the findings. Therefore, the present findings should be considered hypothesis-generating.

In conclusion, even if the present data require confirmation in larger, controlled studies, they provide a hypothesis-generating observation suggesting that dose fractionation may modify the peak-to-pre-dose biochemical pattern observed under once-daily transdermal testosterone administration. Prospective pharmacokinetic studies with standardized full 24-hour sampling are required to clarify whether these observations translate into differences in overall daily testosterone exposure.

## Data Availability

Original data generated and analysed during this study are included in this published article.
